# Caregiver-perpetrated violence against patients with severe mental disorders and associated factors in Bahir Dar, northwest Ethiopia

**DOI:** 10.1016/j.heliyon.2025.e42861

**Published:** 2025-02-19

**Authors:** Mastewal Aschale Wale, Fikir Addisu, Habte Belete, Wubalem Fekadu, Agmas Wassie, Aklile Tsega Chekol

**Affiliations:** a Department of Nursing, College of Medicine and Health Sciences, Hawassa University, Hawassa, Ethiopia; b Department of Psychiatry, College of Medicine and Health Sciences, Bahir Dar University, Bahir Dar, Ethiopia; c Dr. Ambachew Memorial Hospital, South Gondar Zone, Tach Gaynt, Ethiopia

**Keywords:** Caregiver violence, Human rights, Severe mental illness, Ethiopia

## Abstract

**Background:**

Caregiver abuse has widespread negative effects on patients with serious mental illnesses in terms of ethics, law, society, culture, and economy. However, studies in low-income nations, such as Ethiopia, are scarce. Consequently, the purpose of this study was to evaluate the prevalence of caregiver aggression and related variables in patients with severe mental illnesses.

**Methods:**

An institutional-based cross-sectional study was conducted among 394 people with severe mental disorders who were selected through a systematic random sampling technique. Data were gathered through in-person interviews using Epicollect-5 software on a smartphone. The data were then exported into the Statistical Package for Social Science for analysis. A p-value of less than 0.25 in the bivariate analysis indicated a potential candidate for additional multiple logistic regression analysis to control the possible confounders. A p-value of less than 0.05 was used to establish statistical significance in multivariate logistic regression analysis.

**Results:**

The study's participant response rate was 98 %. There was 36.3 % (95 % CI = 31.5 %, 41.3 %) caregiver violence. Of these, 49.7 % (71/143) of them reported having experienced abuse from caregivers in the previous year. A significant number of factors were associated with the outcome variable including, average monthly income of less than 2000 Ethiopian Birr (AOR = 3.31; 95 % CI 1.33, 8.24), co-morbid illness (AOR = 3.45; 95 % CI 1.51, 7.98), number of episodes (AOR = 3.29; 95 % CI 3.59, 11.02), length of illness (AOR = 4.17; 95 % CI 2.19, 7.91), and having a history of aggression in the past (AOR = 4.80; 95 % CI 3.48, 7.90).

**Conclusion:**

More than a third (36.3 %) of individuals with serious mental illnesses experienced caregiver violence. The following factors were found to be predictive of carer violence: income, co-morbid illness, number of episodes, and history of aggression. This indicates there is a need for public health attention among patients with severe mental disorders to prevent the violation of their human rights.

## 1 Introduction

The World Health Organization (WHO) defines violence as the deliberate use of physical harm against others or oneself in self-harm, collective, and interpersonal violence [1]. Experiences of violence, defined as the direct experience of intentional physical harm by others, are devastating to the family, costly to service, and pose complex administrative challenges for clinicians and caregivers [2]. This includes physically beating patients, sexually assaulting them, and robbing them of essentials such as diet, physical care, and medical care [3]. Caregiver violence is an important issue in recovering from mental disorders. It has universal ethical, legal, socio-cultural, economic, and health consequences among patients with severe mental disorders [2].

Severe mental disorder (SMD) is defined as a cognitive, behavioral, or emotional disorder resulting in serious functional impairment that substantially interferes with or limits one or more major life activities. It includes, mainly, schizophrenia and spectrum disorders, bipolar disorder, and major depressive disorder, which are conditions that tend to be chronic and relapsing in nature and may lead to serious impairment in one or more areas of functioning [4]. Exposure of people with SMD to violence can be seen as a reflection of societal stigma towards people with mental illness, and victimization can be seen as an important negative consequence of mental health stigma [5]. The perpetrators of the abuse are mainly the caregivers, including their spouses, in-laws, parents, relatives, and others, in descending order. About a quarter of chronic illness patients reported facing sexual abuse by their spouses during their illness, of these (92 %) accounted for physical abuse by a family member that is, (abused by a husband or ex-husband (51 %), father or stepfathers (40 %), and mothers or stepmothers (23 %)) [1]. Patients with SMD are at higher risk of being victims of all forms of violence than the general population, with a range of 15–45 % [6].

Both the WHO and the World Psychiatric Association have highlighted caregiver violence are a major determinant of mentally ill people [7]. There is no doubt about the evidence from high, middle, and low-income nations: exposure to violence raises the possibility of mental health problems. The psychological consequences of carer violence can have a detrimental impact on patients' lives. These consequences include depression, anxiety, PTSD, psychosis, self-harm, sexual issues, unreliability of others, and a range of risky and psychosomatic behaviors [8]. In addition, a variety of adult psychopathologies, such as those that impact mood, anxiety, and behavior, can be exacerbated by early exposure to violence [9]. It includes physical, sexual, and emotional violence, which is sadly highly prevalent, general practitioners and mental health professionals need to routinely ask people with mental health problems about current and historical violence and abuse [7]. According to one study, patients who had experienced violent events had lower scores for the environmental quality of life domain and lower social function scores [10].

The violence experienced by people with SMD is associated with reduced symptomatic treatment, functional recovery, and reduced treatment adherence [11,12]. It is also associated with suicide attempts, depression [13,14], post-traumatic stress disorder, and increased alcohol consumption [15]. Caregiving is a time-consuming responsibility that causes caregivers social, emotional, behavioral, and financial problems, as well as limiting their personal lives. Furthermore, it may have an impact on their ability to care for mentally ill relatives and though patients need quality care usually attendants will abuse patients which will aggravate their illness and will result in a relapse of the illness [16]. People with SMD are generally considered to be more likely to perpetrate violence than the general population; however, evidence of significant levels of physical victimization is increasingly recognized [17,18].

According to research, patients in mental health services are up to eleven times more likely than the general population to have experienced recent violence. Based on the findings of a previous study, more than one-quarter of persons with severe mental disorders had been victims of violence [19]. Persons with pre-existing SMD are at significantly higher risk of being victims of all forms of violence than the general population, with victimization rates ranging from 15 to 45 % in the past twelve months, and 40–90 % over a lifetime [18]. Caregivers sometimes insist patients take medications and report patients' psychopathology as triggers for violent behavior that should be managed by talking to the patient calmly and lovingly [20]. However, with the presence of caregivers at the assessment and low levels of privacy patients may not report experiencing violence from their caregivers [5].

In India, out of 406 patients, 65 % suffered from some form of violence with 52 % suffering from emotional violence, 27.6 % from physical violence, and 24.8 % from sexual violence [1]. The most common types of violence reported among female patients were emotional abuse (75 %), physical violence (51 %), sexual violence (17 %), and economic abuse in the form of economic deprivation (43 %) [1]. Research from several countries revealed that the prevalence of caregiver violence was 19.8 % in Spain (physical, emotional, and sexual violence rates were 10.7 %, 17.7 %, and 3.2 %, respectively) [21], 18.9 % in China [10], and 79.7 % in Istanbul, Turkey had been subjected to violence by their partners [22].

In Ethiopia, a cross-sectional study has shown that patients with bipolar disorder experience high levels of physical and verbal violence from their caregivers. The percentage of people who reported physical or verbal violence was 37.7 %. In terms of the type of violence, 25.3 % of the participants reported physical violence, 32.6 % reported insults, 31.4 % were reprimanded, 10.2 % were sent away at meals, 33.6 % were shouted at by caregivers, and 12.9 % were barred from participating in family activities [23]. Data are scarce on the context and character of caregiver violence, risk factors, and responses [24] and there is a limited study in low-income countries including Ethiopia. Therefore, this study aimed to assess the proportion of caregiver violence and associated factors among patients with severe mental disorders.

## 2 Methods and materials

An institutional-based cross-sectional study was conducted from June 18 to July 16, 2022, in Bahir Dar City's two hospitals. Bahir Dar is the capital of the Amhara regional state in northwest Ethiopia, about 490 km from Addis Ababa. It is one of the leading and best tourist destinations in Ethiopia, with a variety of attractions near Lake Tana and the Blue Nile River.

Both Tibebe Ghion Specialized Hospital (TGSH) and Felege Hiwot Specialized Comprehensive Hospital (FHCSH) are found in Bahir Dar, Amhara Regional State. These hospitals offer both inpatient and outpatient mental health services. There are 13 beds in TGSH and 17 beds in FHCSH. The number of annual outpatient clients is 4864 (405 per month) in TGSH and 19,200 (1600 per month) in FHCSH.

All people with SMD who have follow-up visits at TGSH and FGCSH were the source population, and the study population was people with SMD who have follow-up visits during the data collection period. People with SMD older than 18 years who had at least three months of follow-up treatment in these hospitals were included and those who are seriously ill (patients who have communication problems due to psychopathology and who are in an unstable condition) were excluded. The final sample size of the study was 398.

On average there was 345 and 645 patients at TGSH and FHCSH respectively per month. The sampling interval (K) was determined by N/n = 990/398, which was approximately 3. The data was collected at three intervals until the desired sample size was reached. The proportional allocation for both hospitals was 1:2 (345/645) and 133 patients from TGSH and 265 patients from FHCSH were included. The dependent variable was caregiver violence and socio-demographic variables (age, sex, religion, residence, occupation, marital status, average monthly income of the household, educational status), clinical factors (type of mental illness, duration of the illness, number of episodes, family history of mental illness, comorbid illness, previous aggressive behavior) and psychosocial factors (social support, substance use, caregiver relationship, stigma) were the independent variables.

The data was collected by using a questionnaire adapted from related literature and caregiver violence was measured by using an interviewer-administered standardized checklist adapted from the National Crime Victimization Survey Basic Screen Questionnaire (NCVS). The domains that fit with our study population (physical violence) were used [25] and emotional violence was measured by a WHO multi-culturally validated questionnaire. This measuring tool has been utilized in previous studies to assess the magnitude of violence among mentally ill patients [1]. The NCVS instruments have several strengths: they allow the comparison of the data with general population, they are the most comprehensive instruments available to assess victimization, and they have been extensively tested [26]. The NCVS has 2 parts: 1. The Basic Screen is a brief instrument eliciting demographic information and identifying the number and types of possible violence to explore. For example, the screen asks respondents (specifying the recall period): “Have you ever been attacked or threatened?” 2. The Crime Incident Report then elicits detailed information on each event. These detailed data allow the researcher to determine whether each event is a crime, when it occurred, who was involved if the police were notified. Because the NCVS is designed for use in the general population, we modified the wording of the survey to fit the needs of our sample. Oslo social support scale measured the level of social support. **It** is a three-item scale with Cronbach alpha of 0.75 and has a range value of 3–14, further categorized as follows: “poor support,” 3–8; “moderate support,” 9–11; and “strong support,” 12–14 [27]. The stigma scale for chronic illness was developed and validated by Molina et al. It is a short, reliable, and valid instrument to assess the impact of stigma [28]. The SSCI-8 has eight questions that assess internalized and enacted domains of stigma at the personal dimension. Responses on the SSCI-8 were scored from 1 to 5 for each item, where 1 = never, 2 = rarely, 3 = sometimes, 4 = often, and 5 = always with a score range of 8–40.

The questionnaire was prepared first in English and then translated into the Amharic language for the better understanding of the data collectors and the respondents. The data were collected by three BSc psychiatry nurses. The quality of data was ensured through training for one day before the actual data collection for data collectors on the objective of the study, data collection tools and procedures, and how to keep confidentiality. Pre-test was done two weeks before the actual data collection at Addis Alem Hospital on 20 patients (5 % of the total sample size); the result was not included in the main study. The result of the pre-test shows good validity with Cronbach's alpha of (0.870). The final data collection tool was refined based on the findings from the pre-test. Data was collected by Epicollect-5 software application using a smartphone and then exported to Statistical Package for Social Science (SPSS) version 25 for analysis. Descriptive statistics like frequencies, standard deviation, percentages, and means were used to characterize study subjects. The results were presented using charts, graphs, and tables. Binary logistic regression has been used to identify factors associated with caregiver violence. A bivariate binary logistic regression analysis with a cutoff point P-value ≤0.25 was used to identify possible candidate predictors for the entire model [29]. Statistical significance was declared at a p-value less than 0.05 in multivariate logistic regression analysis with the outcome variable. The model was tested in multivariate logistic regression and the value of the Hosmer and Lemeshow goodness of fit test result was 0.639, with no multicollinearity (Tolerance>0.1 and Variance inflation factor (VIF) < 3). An odds ratio with 95 % confidence intervals was used to show the strength of the association.

The study was approved by Institutional Review Board (IRB) of Bahir Dar University College of Medicine and Health Sciences with this reference number (487/2022) before the actual data collection and a permission letter was obtained from the Research Directorate office of Bahirdar University and was given to TGSH and FHCSH. Oral informed consent was obtained from each study participant. Participants who were given oral consent were eligible to participate in the study and confidentiality was assured by data collectors and supervisors.

## 3 Results

### 3.1 Socio-demographic characteristics

From the total 398 questionnaires, 394 questionnaires were filled resulting in a response rate of 98 %. Of the total participants, 52.3 % were females. The mean age of the participants was 33.23 years (SD 33.23 ± 10.289) (Table 1).

**Table 1:** Distribution of demographic characteristics of people with SMD at TGSH and FHCSH, in 2022 (n = 394).

Variables	Categories	Violence	
		Yes (%)	No (%)
Sex	Male	50.3 %	46.6 %
	Female	49**.7 %**	53.4 %
Age	18–39	72.0 %	75.3 %
	40–65	27.3 %	23.9 %
	>65	0.7 %	0.8 %
Residence	Urban	39.9 %	58.2 %
	Rural	60.1 %	41.8 %
Marital status	Single	37 %	30.5 %
	Married	37.1 %	49.8 %
	Divorced/widowed	25.9 %	19.7 %
Religion	Orthodox	69.2 %	67.7 %
	Muslim	26.6 %	25.9 %
	Protestant	4.2 %	6.4 %
Ethnicity	Amhara	97.9 %	97.6 %
	∗∗∗Others	2.1 %	2.4 %
Educational status	No formal education	32.9 %	31.1 %
	Primary education	18.9 %	19.1 %
	Secondary education	31.5 %	28.7 %
	Diploma and above	16.8 %	21.1 %
What is your average monthly income in ETB	≤2000	38.1 %	33.5 %
	2001–4000	21.1 %	21.9 %
	4001–6000	21.1 %	22.3 %
	≥6001	19.8 %	22.3 %
Occupation	Farmer	16.8 %	18.3 %
	Government employee	44.8 %	35.9 %
	Private	3.5 %	12.0 %
	Student	25.2 %	25.9 %
	∗Others	9.8 %	8.0 %
Currently living with	Parents	40.6 %	42.2 %
	Spouse	34.3 %	41.8 %
	Sibling	16.8 %	8.0 %
	∗∗Others	8.4 %	8.0 %

∗ = housewife, daily laborer, ∗∗ = Brother and sister, alone, relative, and ∗∗∗ = SNNPR.

### 3.2 Psychosocial and clinical-related factors

Of the total participants (n = 394), 28.7 % had a history of substance use in their lifetime, and from those 17.8 % reported alcohol consumption. In this study, caregivers who mostly made violence towards people with SMD were spouses (36.4 %). Of the total participants, 35 % have poor social support. Of the total participants, 39.1 % were schizophrenic, and 13.5 % had a comorbid illness (Table 2).

**Table 2:** Psychosocial and clinical related factors of caregiver violence among patients with severe mental illness at TGSH and FHCSH, in 2022 (n = 394).

Clinical-related factors	Categories	Violence	
		Yes (%)	No (%)
Type of Mental illness	Schizophrenia	40.6 %	38.2 %
	Bipolar	19.6 %	25.5 %
	MDD	36.4 %	26.7 %
	∗Other psychotic disorder	3.5 %	9.6 %
Duration of illness	≤12 months	18.9 %	54.6 %
	>12 months	81.1 %	45.4 %
Number of episodes	First episode	24.5 %	73.3 %
	Two or more	75.5 %	26.7 %
Previous aggressive behavior	Yes	35.7 %	9.2 %
	No	64.3 %	90.8 %
Family history of mental illness	Yes	22.4 %	21.5 %
	No	77.6 %	78.5 %
Comorbid illness	Yes	19.6 %	10.0 %
	No	80.4 %	90.0 %
Social support	Poor	41.3 %	31.5 %
	Moderate	30.8 %	30.7 %
	Strong	28.0 %	37.8 %
Relationship (mostly abuser)	Spouse	36.4 %	70.5 %
	Father	21.0 %	7.8 %
	Brother/sister	23.8 %	12.7
	∗∗others	18.9 %	9.2 %
Substance use	Yes	48.3 %	17.5 %
	No	51.7 %	82.5 %
Type of substance	Alcohol	81.8 %	93.2 %
	Khat	16.1 %	5.6 %
	Cigarette	2.1 %	1.2 %

∗ = schizoaffective, schizophreniform, brief psychotic disorder, MDD = Major Depressive Disorder.

∗∗ = mother, sibling, uncle.

### 3.3 The magnitude of caregiver violence

**Fig. 1:**
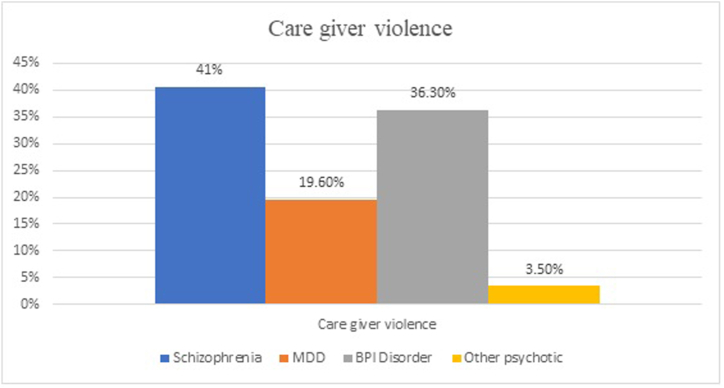
Caregiver violence on severe mental illness.

In this study, the overall magnitude of caregiver violence was 36.3 % (95 % CI = 31.5 %, 41.1 %). Based on the type of violence, 30.7 % had emotional violence, and 28.7 % had physical violence. Of the participants who were exposed to caregiver violence (n = 143), 50.3 % were females and 60.1 % lived in a rural area. Of the total participants who have caregiver violence, 44 % reported caregiver violence in the past twelve months. Of the total participants, 72.7 % reported that they faced caregiver violence two or more times in their lifetime and 58.4 % had a duration of more than a year. Of the total participants, 48.25 % have a history of lifetime substance use and 35.7 % have previous aggressive behavior (Fig. 1).

Regarding emotional and physical violence, belittled or humiliated you in front of other people and any attack or threat or use of force by anyone at all accounted 81.2 % and 21.8 %, respectively (Table 3).

**Table 3:** Distribution of emotional and physical violence in people with severe mental disorders who have follow-up treatment at TGSH and FHCSH, in 2022 (n = 394).

Questions	Responses	
Emotional violence	Yes	No
Insulted you or made you feel bad about yourself?	27.7 % (109)	72.3 % (285)
Belittled or humiliated you in front of other people?	81.2 % (320)	18.8 % (74)
Did things scare or intimidate you on purpose (by the way he/she looked at you, by yelling and smashing things)?	18.8 % (74)	81.2 % (320)
Threatened to hurt you or someone you care about?	12.4 % (49)	87.6 % (345)
Physical violence		
Did they use any weapon, for instance, a gun or knife	3.3 % (13)	96.7 % (381)
With anything like hot things, scissors, or stick	11.4 % (45)	88.6 % (349)
Did they throw something at you, such as a rock or bottle	9.9 % (39)	90.1 % (355)
Did they grab you, punch you, or chock you	15.7 % (62)	84.3 % (332)
Any face-to-face threats	15.2 % (60)	84.8 % (334)
Any attack or threat or use of force by anyone at all? Please mention it even if you are not certain it was a crime.	21.8 % (86)	78.2 % (308)
Have you ever reported the violence to the police?	1.0 % (4)	99.0 % (390)

### 3.4 Factors associated with caregiver violence

In the bivariable logistic regression analysis, nine variables such as residence, occupation, average monthly income, type of illness, duration of illness, social support, family stigma, number of episodes, and comorbid illness were found to be statistically significant with caregiver violence. However, in the multivariate logistic regression, only monthly income, duration of illness, comorbid illness, and number of episodes showed statistical significance with caregiver violence **(**Table 4).

**Table 4:** Bivariate and multivariate logistic regression analysis showing an association between caregiver violence and associated factors among people with SMD, in 2022 (n = 394).

Variables	Categories	Caregiver violence		COR (95 % CI)	AOR (95 % CI)
		Yes	No		
Residence	Rural	86	105	2.10 (1.38, 3.19)	1.87 (3.06, 3.65)
	Urban	57	146	1	1
Occupation	Farmer	64	90	1.02 (0.48, 2.16)	0.72 (0.27,1.90)
	Private	36	65	0.79 (0.36, 1.75)	1.40 (0.57,3.47)
	Student	24	46	0.75 (0.32, 1.73)	0.65 (0.24,1.78)
	Other	19	50	1	1
Average monthly income	**<2000**	**66**	**84**	**2.00 (1.11, 3.61) ∗**	**3.31(1.33,8.24) ∗∗**
	2001–4000	28	55	1.30 (0.66, 2.54	2.24 (0.87,5.79)
	4001–6000	27	56	1.23 (0.63, 2.41)	1.57 (0.62,3.98)
	>6001	22	56	1	1
Type of mental illness	Psychotic	63	120	1.20 (1.70, 8.06)	1.38 (0.35,5.49)
	BPI disorder	52	67	1.77 (1.00, 3.15)	2.78 (0.69,11.29)
	MDD	28	64	1	1
Duration of illness	**>12 months**	**116**	**114**	**5.16(3.17, 8.40) ∗**	**4.17(2.19,7.91) ∗∗**
	≤12 months	27	137	1	1
Episode	**Two or more**	**108**	**67**	**8.47 (5.28, 13.60) ∗**	**3.29(3.59,11.02) ∗∗**
	1st episode	35	184	1	1
Comorbid illness	**Yes**	**28**	**25**	**2.20 (1.23, 3.95) ∗**	**3.45(1.51,7.98) ∗∗**
	No	115	226	1	1
Social support	Poor	59	79	1.77 (1.08, 2.93)	1.181 (0.58,2.40)
	Moderate	44	77	1.36 (0.80, 2.29)	1.04 (0.51,2.14)
	Strong	40	95	1	1
Family stigma	Yes	18	66	0.40 (0.23, 0.71)	0.54 (0.26, 1.13)
History of previous aggression	No **Yes** No	125 **51** 92	185 **23** 228	1 **5.49(3.18,9.51)** ∗ 1	1 **4.80(3.48,7.90) ∗∗**

∗ = P < 0.25, ∗∗ = P < 0.05, AOR = Adjusted Odds Ratio, COR=Crude Odds Ratio, MDD = Major Depressive Disorder, BPI = bipolar disorder, SMD=Severe Mental Disorder, 1 = reference group.

## 4 Discussion

In this study, the proportion of caregiver violence was 36.3 % with (95 % CI = 31.5 %, 41.1 %). The current study (36.3 %) is in line with a study conducted in Ethiopia 37.7 % [23] in Amanuel Mental Specialized Hospital. However, the proportion of caregiver violence in the current study is lower than study findings from other studies conducted in India 65 % [1]. The likely explanation could be in the previous studies both new and follow-up cases were included; however, in the current study patients with follow-up treatment for at least three months were included to prevent bias. The other reason might be they use a self-administered questionnaire but we used an interviewer-administered questionnaire. On the other hand, the proportion of caregiver violence in the current study is higher than the study finding done in Spain among people with mental illness which was 19.8 % [21]. The possible explanation for this difference might be due to differences in measuring tools, methodological differences, and sociocultural differences among the study participants.

In the final model of multiple logistic regression analysis, average monthly income (<2000 ETB), having the comorbid illness, the number of episodes (two or more), duration of illness of more than a year, and history of previous aggression showed statistical significance with caregiver violence. The odds of having caregiver violence among participants who have comorbid illness was threefold increased as compared to participants with no comorbid illness (AOR = 3.45; 95 % CI 1.51, 7.98**)**. The possible reason could be having additional comorbid illness increases the burden on caregivers, and patients might be highly exposed to caregiver violence. Previous studies showed that some disorders can cause behavioral disturbances and may lead patients to develop irritable and aggressive behavior that triggers their caregiver to violence [23]. Violence may also be sparked by having more medical expenses, illnesses, or behavioral issues that present a challenge for caretakers. The odds of having caregiver violence among respondents who have two or more episodes were three times higher as compared to those who have only one episode (AOR = 3.29; 95 % CI 3.59, 11.02). This might be due to the episodic nature of mental illness insists caregivers experience violence towards mentally ill patients due to increased burden. Moreover, it could be due to costing additional admission or time to care for each episode. The other possible rationale is recurrences of psychopathology result in difficult behaviors that distress the caregivers, leading them to be aggressive toward the patients [30]. People with SMD are at risk for several psychosocial disadvantages due to the episodic nature of their illness, including unemployment, strained social relationships, and excessive medical costs. Conflicts in the patient's environment brought by this psychosocial deficit may encourage caregiver violence. Patients who experience frequent episodes of illness face a variety of difficulties, including being exposed to violence [31].

The odds of having caregiver violence among participants who have an average monthly income of less than 2000 ETB is threefold higher as compared to those who have an average monthly income of greater than 6001 ETB (AOR = 3.31; 95 % CI 1.33, 8.24**)**. This could be due to caregivers' financial constraints and higher treatment costs, which contribute to neglect and violence [32]. This is further confirmed by the fact that the bulk of caregiver violence occurred excessively among low-income people. This demonstrates how the expensive cost of treatment can lead to a perpetuation of violence [1]. Previous studies showed that the caregiver's level of income was reported to negatively affect the economic burden experienced by caregivers and this may expose people with SMD to caregiver violence [33]. The possible rationale could be that they spend much of their time caring for the patients, taking them to holy water treatments that are far from their village. This depleted their financial resources, which irritated them and triggered caregiver violence toward the patients.

The odds of having caregiver violence were fourfold higher in patients with a duration of illness of more than one year as compared to a duration of illness of less than a year (AOR = 4.17, 95 % CI = 2.19,7.91). This was supported by previous studies conducted in Ethiopia among people with SMD attending outpatient clinics [34]. The duration of illness studies showed that caregivers of people with a longer duration of illness experienced more burdens and this may trigger caregiver violence toward people with SMD [35]. As the disease lasted longer, the caregiver began to feel worthless and irritable about trivial matters because they thought they couldn't heal their illness. The odds of having caregiver violence among patients who have a history of previous aggression during their morbidity were four times higher as compared to no history of previous aggression (AOR = 4.80; 95 % CI 3.48,7.90). This might be due to the violent behaviors of patients towards their caregivers and other persons; their caregivers may violate them to prevent them from harm. As studies showed that, the most likely targets of violence were family members or close friends (87 %) [36], caregivers may practice violence towards patients with mental illness either intentionally or due to a lack of understanding of the nature of the illness. People with SMD with a duration of illness of more than a year were associated with caregiver violence.

## 5 Limitations of the study

Being a cross-sectional study design hence, it did not show the causal effect relationship. Another limitation is that the measuring tool used to assess caregiver violence was not validated in our country cultural context.

## 6 Conclusion

Caregiver violence was more than a third (36.3 %) among people with severe mental disorders. Income, co-morbid illness, the number of episodes, duration of illness, and history of previous aggression were factors significantly associated with caregiver violence. This indicates there is a need for public health attention among patients with severe mental disorders to prevent the violation of their human rights.

## CRediT authorship contribution statement

**Mastewal Aschale Wale:** Writing – review & editing, Writing – original draft, Investigation, Formal analysis, Data curation, Conceptualization. **Fikir Addisu:** Writing – review & editing, Supervision. **Habte Belete:** Writing – review & editing, Supervision. **Wubalem Fekadu:** Writing – review & editing, Supervision. **Agmas Wassie:** Writing – original draft, Writing – review & editing. **Aklile Tsega Chekol:** Writing – review & editing, Writing – original draft, Formal analysis.

## Consent for publication

Not applicable.

## Availability of data statement

Data will be available on request by the corresponding author.

## Funding

There is no special funding for this study.

## Declaration of competing interest

The authors declare that they have no known competing financial interests or personal relationships that could have appeared to influence the work reported in this paper.

## Abbreviations

AOR: Adjusted Odds Ratio
BPI: Bipolar Disorder
CI: Confidence Interval
COR: Crude Odds Ratio
ETB: Ethiopian Birr
FHCSH: Felege Hiwot Specialized Comprehensive Hospital
MDD: Major Depressive Disorder
SD: Standard Deviation
SMD: Severe Mental Disorder
TGSH: Tibebe Ghion Specialized Hospital
WHO: World Health Organization
