# Supplementary material for: Caregiver-perpetrated violence against patients with severe mental disorders and associated factors in Bahir Dar, northwest Ethiopia
Source: Heliyon. 2025 Feb 19;11(4):e42861. doi: 10.1016/j.heliyon.2025.e42861 (PMC11904567; doi:10.1016/j.heliyon.2025.e42861)
Supplement: Multimedia component 1 [file mmc1.docx]

Part I: Socio demographic Characteristics of patient respondents.

Instruction: For each of the following questions please circles the alternative that fit for respondent’s response.

| **Q.No** | **Question** | **Alternative choices** |
| --- | --- | --- |
| 101 | Sex | 1. 1. Male 2. Female |
| 102 | Your age? | -------- |
| 103 | Place of Residence | 1. 1. Urban 2. Rural |
| 104 | Your religion? | 1. 1. Orthodox 2. Muslim 2. 3. Protestant 4. Catholic 3. 5. Others, specify______ |
| 105 | What is your current marital status? | 1. 1. Single 2. Married 2. 3. Divorced 4. Widowed |
| 106 | Educational Status | 1. 1-5th grade 2. 5-10th grade  3. Diploma and above |
| 107 | To which ethnic group do you belong? | 1. 1. Amhara 2. Oromo 2. 3. Tigray 4. Sidama 3. 5. SNNRP 4. 6. Others, specify------- |
| 108 | With whom are you currently living? | 1. Parents 2. Sibling 3. Spouse 4. brother? Sister 5. other specify |
| 109 | Average Monthly income of the household in ETB | ___ |
| 110 | Full name of the diagnosis | To be filled from patient card  --------------------------- |
| 111 | Duration of illness | ----------------------- |
| 112 | Number of episodes of the illness | 1. 1. First 2. Two or more |
| 113 | Family history of mental illness | 1. 1. Yes 2. No |
| 114 | Do you have any comorbid illness? | To be filled from patient chart ---- |
| 115 | Was there any aggressive behaviour during the time of your morbidity? | 1. Yes 2. No |
| 116 | Have you ever been used any substance? | 1. Yes 2. No |
| 117 | If yes which substance you use? | 1. Alcohol 2. Khat   3. Cigarette  4. Other specify--- |

|  | | **Part** **II**- Caregiver violence questionnaire (WHO and NCVS) | | | | | | | |
| --- | --- | --- | --- | --- | --- | --- | --- | --- | --- |
| **201.** | | I am now going to ask you about some violence that are experienced by many patients with mental illness by their caregivers (may be biological parents, siblings, brother, sister or others who take care of you). It is important to hear from people themselves if we are to understand the serious problem of violence in the home. I’m going to ask you some questions and I’d like you to tell me whether your caregiver has done any of the following to you. Your responses are important whether or not you have had any of these experiences. Remember that all information provided is strictly confidential. I would again assure you that your answers will be kept secret, and that you do not have to answer any questions that you do not want to. May I continue? | | | | | | | |
|  | | The next questions are about things (Emotional violence) that happen to many patients with mental illness that may perform by any person who take care of you. | | | | | | | |
|  | | I want you to tell me if anyone from your caregiver (Does/did) ever do any of the following things to you? Or have you ever having been ----- | | | | | | | |
| A | | Insulted you or made you feel bad about yourself? | | | | 1. 1. Yes 2. No | | | |
| B | | Belittled or humiliated you in front of other people? | | | | 1. 1. Yes 2. No | | | |
| C | | Did things to scare or intimidate you on purpose (e.g.by the way he/she looked at you, by yelling and smashing things)? | | | | 1. 1. Yes 2. No | | | |
| D | | Threatened to hurt you or someone you care about? | | | | 1. 1. Yes 2. No | | | |
| **202.** | | The next questions are about things that happen to many patients with mental illness that may perform by any person who take care of you. | | | | | | | |
|  |  | Has anyone of your caregiver attacked or threatened you in any of these ways? | | | | | | | |
| A | | Did they use any weapon, for instance, a gun or knife | | | | 1. 1. Yes 2. No | | | |
| B | | With anything like, hot things, scissors, or stick | | | | 1. 1. Yes 2. No | | | |
| C | | Did they throw something at you, such as a rock or bottle | | | | 1. 1. Yes 2. No | | | |
| D | | Did they grab you, punch you, or chock you | | | | 1. 1. Yes 2. No | | | |
| F | | Any face-to-face threats | | | | 1. 1. Yes 2. No | | | |
| G | | Any attack or threat or use of force by anyone at all? Please mention it even if you are not certain it was a crime. | | | | 1. 1. Yes 2. No | | | |
| H | | How many times? | | | | 1. 1. Once 2. Two times 2. 3. >3times 4. Don’t know | | | |
| I | | Who is the one that mostly abuse you among your caregivers (both emotional and physically)? | | | | 1. 1. Mother 2. Father 3. Spouse 2. 4. Sibling 5. Brother 6. Sister 3. 7. Other specify | | | |
| J | | When it occurred? | | | | 1. During last 12 months 2. Before 1 year | | | |
| L | | Have you ever reported the violence to the police? | | | | 1. 1. Yes 2. No | | | |
| **Part III. The Oslo 3-items social support scale** | | | | | | | | | |
| 301 | | How easy can you get help from neighbors if you should need it? | Very easy | Easy | | Possible | Difficult | Very Difficult |  |
| 302 | | How many people are so close to you that you can count on them if you have serious problems? | None | 1-2 | | 3-5 | 5+ |  |  |
| 303 | | How much concern do people show in what you are doing? | A lot | Some | | Uncertain | Little | No |  |

| **Part IV**. Medication Adherence   \| 401 \| \| Do you sometimes forget to take your prescribed medicines? \| \| --- \| \| \| \| \| 1. Yes \| \| 1. No \| \| \| --- \| --- \| --- \| --- \| --- \| --- \| --- \| --- \| --- \| --- \| \| 402 \| Over the past 2 weeks, were there any days when you did not take your prescribed medicines? \| \| \| \| 1. Yes \| \| 1. No \| \| \| 403 \| Have you stopped taking medications because you feel worse when you take it? \| \| \| \| 1. Yes \| \| 1. No \| \| \| 404 \| When you travel or leave home, do you sometimes forget to bring along your meds? \| \| \| \| 1. Yes \| \| 1. No \| \| \| 405 \| Did you take your prescribed medicine yesterday? \| \| \| \| 1. Yes \| \| 1. No \| \| \| 406 \| When you feel like your health is under control, do you sometimes stop taking your meds? \| \| \| \| 1. Yes \| \| 1. No \| \| \| 407 \| Do you feel hassled about sticking to your prescribed treatment plan \| \| \| \| 1. Yes \| \| 1. No \| \| \| 408 \| How often do you have difficulty remembering to take all your prescribed medicine? \| \| \| \| Never/ Rarely (1)  Once in a while (0)  Sometimes (0)  Usually (0) \| \| \| \| \| **Part V: SSCI-8 (**Family stigma) \| \| \| \| \| \| \| \| \| \| \|  \| \| 1=never \| 2=rarely \| 3=sometimes \| \| 4=often \| \| 5=always \| \| \| Because of your illness, some of your family seemed uncomfortable with you? \| \| 1 \| 2 \| 3 \| \| 4 \| \| 5 \| \| \| Because of your illness, some of your family avoided you? \| \| 1 \| 2 \| 3 \| \| 4 \| \| 5 \| \| \| Because of your illness, some of your family left you out of things? \| \| 1 \| 2 \| 3 \| \| 4 \| \| 5 \| \| \| Because of illness, some family members were unkind to you. \| \| 1 \| 2 \| 3 \| \| 4 \| \| 5 \| \| \| Because of your illness, some family members avoided looking at you? \| \| 1 \| 2 \| 3 \| \| 4 \| \| 5 \| \| \| Some of your family felt embarrassed about your illness \| \| 1 \| 2 \| 3 \| \| 4 \| \| 5 \| \| \| Some of your family felt embarrassed because of your physical limitations \| \| 1 \| 2 \| 3 \| \| 4 \| \| 5 \| \| \| Some family members acted as though it was your fault, you have this illness \| \| 1 \| 2 \| 3 \| \| 4 \| \| 5 \| \|   We have now finished the interview. Thank you. |
| --- | --- | --- | --- | --- | --- | --- | --- | --- | --- | --- | --- | --- | --- | --- | --- | --- | --- | --- | --- | --- | --- | --- | --- | --- | --- | --- | --- | --- | --- | --- | --- | --- | --- | --- | --- | --- | --- | --- | --- | --- | --- | --- | --- | --- | --- | --- | --- | --- | --- | --- | --- | --- | --- | --- | --- | --- | --- | --- | --- | --- | --- | --- | --- | --- | --- | --- | --- | --- | --- | --- | --- | --- | --- | --- | --- | --- | --- | --- | --- | --- | --- | --- | --- | --- | --- | --- | --- | --- | --- | --- | --- | --- | --- | --- | --- | --- | --- | --- | --- | --- | --- | --- | --- | --- | --- | --- | --- | --- | --- | --- | --- | --- | --- | --- | --- | --- | --- | --- | --- | --- | --- | --- | --- | --- | --- | --- | --- | --- | --- | --- | --- | --- | --- | --- | --- | --- | --- | --- | --- | --- | --- | --- | --- | --- | --- | --- | --- | --- | --- | --- | --- | --- | --- | --- | --- | --- | --- | --- | --- | --- | --- | --- | --- | --- | --- | --- | --- | --- | --- | --- | --- | --- | --- |

**በአማርኛ የተዘጋጀ መጠይ**

**ክፍል 1: ማህበራዊ እና የስነ- ህዝብ መረጃ**

መመሪያ፡እባክዎን ቀጥሎ የቀረቡትን ጥያቄዎችና የተሰጡትን አማራጮች ድምፅዎን ከፍ አድርገው በማንበብ የተሳታፊዎቹን ትክክለኛ ምላሽ ያክብቡት።

|  | ጥያቄ | አማራጮች |
| --- | --- | --- |
| 101 | ጾታ | 1. ወንድ 2. ሴት |
| 102 | እድሜዎ ስንት ነው | -------- |
| 103 | መኖርያ ቦታ | 1.ከተማ  2. ገጠር |
| 104 | ሀይማኖትዎ ምንድን ነው? | 1. ኦርቶዶክስ  2. ሙስሊም  3. ፕሮቴስታንት  4. ካቶሊክ  5. ሌሎች፣ ይግለጹ______ |
| 105 | የጋብቻ ሁኔታ? | 1. ያገባ/ች 2. ያላገባ/ች 3. ተለያይቶ/ታ የሚኖር/የምትኖር 4. የተፋታ/ች 5. የትዳር አጋር የሞተበት/ባት |
| 106 | የትምህርት ሁኔታ | 1. 1-5 ኛ ክፍል  2. 5-10 ኛ ክፍል  3. ዲፕሎማና ከዚያ በላይ |
| 107 | ብሄርዎ ምንድን ነዉ? | 1. አማራ 2. ኦሮሞ 3. ትግራይ 4. ሲዳማ 5. ደ/ብ/ብ/ህ ክልል 6. ሌሎች፣ ይግለጹ------ |
| 108 | በአሁኑ ጊዜ ከማን ጋር ነው የሚኖሩት (የሚያሰታምምዎት ማን ነው)? | 1. ከወላጆች  2. ከልጅ  3. ከእህት / ወንድም  3. ከዘመድ  4. ብቻውን  8.ሌላ ይግለጹ ------------ |
| 109 | ወርሃዊ የገቢ መጠን በብር | ------------- |
| 110 | የታካሚው/ዋ ህመም ሙሉ መጠርያ | እባክዎትን ከካርድ ይመለከቱ። |
| 111 | ህመመዎ ለምን ያክል ጊዜ ቆየ? | ------------- |
| 112 | በቤተሰብ የአእምሮ ህመም ያለበት ሰው አለ? | 1. አዎ 2. የለም |
| 113 | ሌሎች ተጓዳኝ ህመም አለብዎት? | እባክዎ ከካረዱ ይመልከቱ  ---------- |
| 114 | በታመሙበት ወቅት የመቆጣት ባህሪ ነበረብዎት? | 1. አዎ 2. የለም |
| 115 | ህመመዎ የዛሬውን ጨምሮ ለምን ያክል ጊዜ ተነሳ/አመመዎት? | 1. አንድ ጊዜ 2. ሁለትና ከዚያ በላይ |
| 116 | ጎጅ/ አደንዛዥ እጽ/መጠጥ ተጠቅመው ያውቃሉ | 1. አዎ 2. የለም |
| 117 | አወ ከሆነ የትኛውን ነው የሚጠቀሙት | 1.አልኮል 2. ጫት 3. ሲጋራ |

|  | | | | **ክፍል 2፡ የተንከባካቢ ጥቃት መጠይቅ** | | | | | | | | | | |
| --- | --- | --- | --- | --- | --- | --- | --- | --- | --- | --- | --- | --- | --- | --- |
| **201.** | | | | አሁን የምጠይቅዎት በብዙ የአእምሮ ሕመምተኞች ላይ ተንከባካቢአቸዉ (ወላጅ፣ ወንድም፣ እህት፣ ልጅ፣ ወይም ሌሎች) ሊያደርሱባቸው ስለሚችሉት አንዳንድ ጥቃቶች ነው። በቤት ውስጥ ያለውን ከባድ የጥቃት ችግር ለመረዳት ከሰዎች ከራሳቸው መስማት አስፈላጊ ነው:: ስለሆነም ተንከባካቢዎ/አስታማሚዎ ከሚከተሉት አንዱን አድርጎብዎት ከሆነ እንዲነግሩኝ እፈልጋለሁ። ከእነዚህ ጥቃቶች ውስጥ አንዳቸውም ቢኖሩዎትም ባይኖሩዎትም የእርስዎ ምላሾች አስፈላጊ ናቸው። ሁሉም የቀረበው መረጃ በጥብቅ ሚስጥራዊ መሆኑን ያስታውሱ። መልሶችዎ በሚስጥር እንደሚጠበቁ እና ለማይፈልጉአቸው ጥያቄዎች መልስ መስጠት እንደሌለብዎ በድጋሚ አረጋግጥልዎታለሁ። ልቀጥል? | | | | | | | | | | |
|  | | | | የሚቀጥሉት ጥያቄዎች የአእምሮ ሕመም ባለባቸው ብዙ ሕመምተኞች ላይ ሊደርሱ ስለሚችሉ **ሥነ-ልቦናዊ** ጥቃቶች ሲሆን እነርስዎን የሚንከባከብ ማንኛውም ሰው ሊያደርጋቸው ይችላል። | | | | | | | | | | |
|  | | | | እርስዎን የሚንከባከቡ ሰዎች የሚከተሉትን ነገሮች አድርሶብዎት ያውቃሉ? ወይስ ከዚህ በፊት ተደርጎብዎት ያውቃል? | | | | | | | | | | |
| ሀ | | | | ስሜት የሚጎዳ ስድብ ወይም ዘለፋ ግጥሞዎት ያውቃል? | | | | | 1. አዎ 2. የለም | | | | | |
| ለ | | | | በሌሎች ሰዎች ፊት የሚያንቐሽሽ/የሚያሸማቅቅ ንግግር ገጥሞዎታል? | | | | | 1. አዎ 2. የለም | | | | | |
| ሐ | | | | በእርስዎ ላይ ተጽእኖ የሚፈጥር ወይም የሚያስፈራራ ድርጊት ( ለምሳሌ፡-ባልሆነ አስተያየት ማየት፣ በመጮህ፣ በመተግበር) ገጥሞዎት ያውቃል? | | | | | 1. አዎ 2. የለም | | | | | |
| መ | | | | በእርስዎ ላይ ተጽእኖ ለመፍጠር፡- በራስዎ ላይ ወይም ለእርስዎ ቅርብ በሆኑ ሰዎች ላይ ጉዳት ለማድረስ የዛቻና ማስፈራሪያ ድርጊት ገጥሞዎት ያውቃል? | | | | | 1. አዎ 2. የለም | | | | | |
| **202.** | | | | የሚቀጥሉት ጥያቄዎች የአእምሮ ሕመም ባለባቸው ብዙ ሕመምተኞች ላይ ስለሚደርሱት ነገሮች(አካላዊ ጥቃት) ሲሆኑ እርስዎን የሚንከባከብ ማንኛውም ሰው ሊያከናውናቸው የሚችሉ ናቸዉ። | | | | | | | | | | |
|  |  |  |  | ከሚከተሉት መንገዶች ውስጥ ከተንከባካቢዎ መካከል ማንኛዉም ሰዉ አጥቅቶዎት ወይም አስፈራርቶዎት ያዉቃል? | | | | | | | | | | |
| ሀ | | | | በማንኛውም መሳሪያ በመጠቀም፣ ለምሳሌ ሽጉጥ ወይም ቢላዋ | | | | 1. አዎ 2. የለም | | | | | | |
| ለ | | | | እንደ መጫዎቻ ዘንግ፣ የጋለ ነገር፣ መቀስ ወይም ዱላ ያለ ማንኛውም ነገር በመጠቀም | | | | 1. አዎ 2. የለም | | | | | | |
| ሐ | | | | ነገሮችን ወርዉረዉብዎት ያዉቃሉ? ለምሳሌ ድንጋይ ወይም ጠርሙስ | | | | 1. አዎ 2. የለም | | | | | | |
| መ | | | | በሀይል መያዝ፣ መምታት ወይም ማነቅ አጋጥሞዎታል? | | | | 1. አዎ 2. የለም | | | | | | |
| ሠ | | | | ማንኛውም ፊት-ለፊት ማስፈራሪያዎች አድርሰዉብዎት ያዉቃሉ? | | | | 1. አዎ 2. የለም | | | | | | |
| ረ | | | | በማንኛውም እርስዎን በሚንከባከብ ሰው የደረሰብዎት ጥቃት ወይም ዛቻ ወይም የኃይል እርምጃ አለ? እባክዎ ወንጀል መሆኑን እርግጠኛ ባይሆኑም ይጥቀሱት። | | | | 1. አዎ 2. የለም | | | | | | |
| ሰ | | | | ምንድን ነው የተፈጠረው? | | | | ----- | | | | | | |
| ሸ | | | | ጥቃቱ የደረሰብዎት መቼ ነበር? | | | | 1. ባለፈዉ 12 ወራት ውስጥ 2. ከ 1 ዓመት በፊት | | | | | | |
| ቀ | | | | ስንት ጊዜ አጋጥሞዎታል? | | | | 1. አንድ ጊዜ 2. 2 ጊዜ 3. > 3 ጊዜ 4. አላውቅም | | | | | | |
| ቀ | | | | በብዛት ጥቃቱን (ስነ-ልቦናዊም ሆነ አካላዊ) የሚያደርስብዎት አካል ማን ነበር? (ከአንድ በላይ መልስ ሊሆን ይችላል). | | | | 1. እናት 2. አባት 3. የትዳር ጓደኛ 4. ልጅ 5. ወንድም 6. እህት 7. ሌላ ይግለጹ | | | | | | |
| በ | | | | ጥቃቱን ለፖለስ አሳውቀዋል? | | | | 1. አዎ 2. የለም | | | | | | |
| **ክፍል 3፡- የኦስሎ ማህበራዊ ድጋፍ መለኪያ መጠይቅ** | | | | | | | | | | | | | | |
| 301 | ከጎረቤትዎ እርዳታ/ድጋፍ ቢያስፈልግዎ ማግኘት ምን ያህል ቀላል ነው? | | | | በጣም ቀላል | | | | | 1 | |  | | |
|  |  |  |  |  | ቀላል | | | | | 2 | |  | | |
|  |  |  |  |  | የሚቻል | | | | | 3 | |  | | |
|  |  |  |  |  | አስቸጋሪ | | | | | 4 | |  | | |
|  |  |  |  |  | በጣም አስቸጋሪ | | | | | 5 | |  | | |
| 302 | ከፍተኛ ችግር ቢያጋጥምዎ የቅርብ የሆኑ እና ይረዱኛል ብለው የሚተማመኑባቸው ምን ያህል ሰዎች ይኖራሉ? | | | | ምንም | | | | | 1 | |  | | |
|  |  |  |  |  | 1-2 | | | | | 2 | |  | | |
|  |  |  |  |  | 3-5 | | | | | 3 | |  | | |
|  |  |  |  |  | 5+ | | | | | 4 | |  | | |
| 303 | ሌሎች ሰዎች ስለ እርሶ ጉዳይ ምን ያህል ግድ ይላቸዋል | | | | በጣም | | | | | 1 | |  | | |
|  |  |  |  |  | የተወሰነ | | | | | 2 | |  | | |
|  |  |  |  |  | አላውቅም | | | | | 3 | |  | | |
|  |  |  |  |  | ትንሽ | | | | | 4 | |  | | |
|  |  |  |  |  | ምንም | | | | | 5 | |  | | |
|  | **ክፍል 4**. የመድሀኒት አወሳሰድ መጠይቅ | | | | | | | | | | |  | | |
| 401 | የታዘዘልዎትን መድሃኒቶች መውሰድ አንዳንድ ጊዜ ይረሳሉ? | | | | | | | 1. አዎ 2. የለም | | | | | | |
| 402 | ባለፉት 2 ሳምንታት ውስጥ በሐኪም የታዘዙትን መድሃኒቶች ያልወሰዱባቸው ቀናት ነበሩ? | | | | | | | 1. አዎ 2. የለም | | | | | | |
| 403 | በሚወስዱበት ጊዜ መጥፎ ስሜት ስለተሰማዎት መድሃኒት መውሰድ አቁመዋል? | | | | | | | 1. አዎ 2. የለም | | | | | | |
| 404 | ሲጓዙ ወይም ከቤት ሲወጡ ፣ አንዳንድ ጊዜ መድሀኒቶችዎን ይዘው መሄድ ይረሳሉ? | | | | | | | 1. አዎ 2. የለም | | | | | | |
| 405 | የታዘዘልዎትን መድሃኒት ትናንት ወስደዋል? | | | | | | | 1. አዎ 2. የለም | | | | | | |
| 406 | ጤንነትዎ ጥሩ እንደሆነ ሲሰማዎት አንዳንድ ጊዜ መድሀኒቶችዎን መውሰድ ያቆማሉ? | | | | | | | 1. አዎ 2. የለም | | | | | | |
| 407 | የታዘዘልዎትን መድሃኒት በታዘዘሎት መሰረት ለመውሰድ እንደከበደዎት ይሰማዎታል? | | | | | | | 1. አዎ 2. የለም | | | | | | |
| 408 | ታዘዘልዎትን መድኃኒቶች በሙሉ አስታውሶ ለመውሰድ ይቸገራሉ? | | | | | | | በጭራሽ/ አልፎ አልፎ(2)  አንድ ጊዜ (1)  አንዳንድ ጊዜ (1)  አብዛኛውን ጊዜ(1)  ሁልጊዜ(1) | | | | | | |
| **ክፍል 5፡ የቤተሰብ መገለል (SSCI-8) መጠይቅ**   1. በፍጹም 2. አልፎ አልፎ 3. አንዳንድ ጊዜ 4.አብዛኛውን ጊዜ 5.ሁልጊዜ | | | | | | | | | | | | | | |
| ጥያቄ | | | | | | አማራጭ መልሶች | | | | | | | | |
| 501. | | | በእርስዎ ህመም ምክንያት አንዳንድ ቤተሰቦችዎ ምቾት አጥተው ነበር? | | | **1** | **2** | | | | **3** | | **4** | **5** |
| 502. | | በህመምዎ ምክንያት አንዳንድ ቤተሰብዎ ይርቁዎት ነበር? | | | | **1** | **2** | | | | **3** | | **4** | **5** |
| 503. | | በህመምዎ ምክንያት አንዳንድ ቤተሰብዎ ከነገሮች ውጪ አድርጎዎታል ? | | | | **1** | **2** | | | | **3** | | **4** | **5** |
| 504. | | በህመምዎ ምክንያት አንዳንድ የቤተሰብ አባላት ለእርስዎ ደግ አልነበሩም? | | | | **1** | **2** | | | | **3** | | **4** | **5** |
| 505. | | በሕመምዎ ምክንያት አንዳንድ የቤተሰብ አባላት እርስዎን ማየት አይፈልጉም ነበር? | | | | **1** | **2** | | | | **3** | | **4** | **5** |
| 506. | | አንዳንድ ቤተሰቦችዎ በእርስዎ ህመም ሃፍረት ይሰማቸዋል ? | | | | **1** | **2** | | | | **3** | | **4** | **5** |
| 507. | | አንዳንድ ቤተሰብዎ በእርስዎ አካላዊ የአቅም ውስንነቶች ምክንያት ይሸማቀቃሉ? | | | | **1** | **2** | | | | **3** | | **4** | **5** |
| 508. | | አንዳንድ የቤተሰብ አባላት ይህ በሽታ የመጣው በእርስዎ ጥፋት እንደሆነ አድርገው ይቆጥሩት ነበር? | | | | **1** | **2** | | | | **3** | | **4** | **5** |

አሁን ቃለ ምልልሱን ጨርሰናል። አመሰግናለሁ:
